# Effect of a nutrition education intervention on caregivers' knowledge, attitudes and practices regarding infant feeding and micronutrient powder use in urban health centers in Maputo, Mozambique

**DOI:** 10.3389/fnut.2026.1833701

**Published:** 2026-06-02

**Authors:** Érica Manuel, Francisco Mbofana, Maria Suzana Bata Maguele, Kátia Mangujo, Maria do Rosário O. Martins

**Affiliations:** 1 Instituto Superior de Ciências de Saúde—ISCISA, Maputo, Mozambique; 2 Global Health and Tropical Medicine, GHTM, LA-REAL, Instituto de Higiene e Medicina Tropical, IHMT, Universidade NOVA de Lisboa, Lisboa, Portugal; 3 Centro Internacsional Para Saúde Reprodutiva, Departamento de Pesquisa, Maputo, Moçambique; 4 Ministério da Saúde -MISAU, Departamento de Nutrição, Maputo, Mozambique

**Keywords:** caregivers, infant and young child feeding, knowledge, attitudes and practices, micronutrient powder, Mozambique, nutrition education, social and behavior change communication

## Abstract

**Background:**

Caregivers' knowledge, attitudes and practices (KAP) play a central role in infant feeding behaviors and the correct use of nutrition interventions such as micronutrient powder (MNP). Although nutrition education and MNP supplementation are routinely delivered through child health services in Mozambique, evidence on changes in caregivers' KAP in urban service settings remains limited. The aim of this study was to estimate the effect of a nutrition education intervention on caregivers' KAP regarding infant feeding and micronutrient powder use in two urban health centers in Maputo, Mozambique.

**Methods:**

A quasi-experimental study was conducted among caregivers of infants aged 6–8 months attending routine child health consultations at two urban primary health centers. One facility implemented reinforced nutrition education, while the other provided standard nutrition education. Caregivers completed a structured KAP questionnaire at baseline and after 6 months of follow-up. Changes in knowledge were analyzed over time and between groups using difference-in-differences analysis. Attitudinal indicators were analyzed at the item level, and practices related to MNP use were assessed descriptively at endline in the intervention group only.

**Results:**

A total of 466 caregivers were enrolled at baseline and 442 completed follow-up. Knowledge scores increased significantly from baseline to endline in both the intervention group (65.0% to 96.7%; *p* < 0.001) and the control group (54.6% to 84.4%; *p* < 0.001). Endline knowledge levels were higher in the intervention group (*p* < 0.001), although the magnitude of improvement did not differ significantly (difference-in-differences: +1.8 pp; *p* = 0.381). Attitudinal indicators improved in the intervention group, whereas several declined in the control group. Difference-in-differences results favored the intervention across attitudinal indicators (all *p* < 0.001). At endline, all caregivers in the intervention group reported offering MNP to their children, with high adherence to daily administration and mostly correct preparation.

**Conclusions:**

Strengthening nutrition education delivered through routine child health services was associated with improved caregivers' KAP related to infant feeding and MNP use. Universal uptake and high adherence to MNP indicate that home fortification can be adopted with counseling. These findings support child health programmes to promote recommended feeding practices and reduce micronutrient deficiencies.

## Introduction

Optimal infant and young child feeding (IYCF) during the first 24 months of life is essential for healthy child growth and development. Inadequate feeding practices contribute substantially to undernutrition, micronutrient deficiencies and adverse health outcomes in early childhood. Appropriate IYCF, including the timely introduction of complementary foods with adequate diversity and continued breastfeeding, is central to meeting nutrient requirements during this critical period. However, despite decades of nutrition programming efforts, suboptimal feeding practices remain common in many low- and middle-income country settings, reflecting persistent gaps in caregivers' understanding and implementation of recommended practices. In such contexts, where young children's diets are often monotonous and low in micronutrient-rich foods, home fortification with micronutrient powder (MNP) has been recommended as an effective strategy to improve the micronutrient quality of complementary foods (1–4).

Caregivers' knowledge, attitudes and practices (KAP) are key determinants of infant feeding behaviors and influence decisions related to the timing and quality of complementary feeding, dietary diversity, and the correct use of nutrition interventions. Changes in caregivers' KAP are widely recognized as important intermediate pathways through which nutrition education interventions influence child feeding behaviors. Studies conducted in sub-Saharan Africa and other low-resource settings have shown that inadequate caregiver knowledge and incorrect beliefs about feeding practices are associated with persistently suboptimal behaviors, even in contexts where health services and counseling are available (1, 2, 5, 6). Nutrition education and behavior change communication are fundamental components of effective child nutrition programmes, with evidence indicating that educational interventions delivered to caregivers can improve IYCF knowledge and feeding practices. Systematic reviews of complementary feeding education interventions have reported positive effects on caregiver behavior and, in some cases, on infant feeding outcomes; however, evidence specifically assessing changes in caregivers' KAP over time remains limited (7, 8).

In addition to education, integrated approaches that combine structured behavior-change communication with strategies to enhance diet quality, such as the use of multiple micronutrient powders (MNP) for home fortification of complementary foods, are recommended by global nutrition stakeholders. Guidelines from the World Health Organization (WHO) and related programming guidance emphasize that MNP should be promoted alongside counseling to ensure correct use and to support sustained behavior change around feeding practices (2, 9, 10). Despite the routine provision of IYCF counseling and micronutrient supplementation in many health systems, empirical evidence evaluating changes in caregivers' KAP over time within routine service delivery settings remains limited. Few studies have assessed KAP as a primary outcome within routine health service delivery settings, especially in urban sub-Saharan African contexts, where access to services does not necessarily translate into optimal infant feeding practices. Although studies from countries such as Uganda, Tanzania, Ethiopia and India suggest that nutrition education and social and behavior change interventions can improve caregiver knowledge and feeding practices, evidence from routine urban service settings in sub-Saharan Africa remains limited (5, 7, 11, 12).

In Mozambique, nutrition education is routinely offered through child health services alongside nutritional interventions, but there is limited evidence on whether these activities translate into measurable improvements in caregivers' KAP related to infant nutrition, complementary feeding and MNP use (13–15). The aim of this study was to estimate the effect of a nutrition education intervention on caregivers' KAP regarding infant feeding and MNP use in two urban health centers in Maputo, Mozambique.

## Materials and methods

### Study design and setting

This quasi-experimental study was conducted in two urban primary health centers in Maputo, southern Mozambique. Mozambique is a low-income country in southeastern Africa, administratively divided into northern, central and southern regions. Maputo, the capital city and Matola District, Maputo Province, both located in the southern region, was selected as the study setting due to its high level of urbanization and comparatively better access to routine maternal and child health services.

The intervention was implemented at Matola C Health Center, located in Matola District, Maputo Province, while 1° de Maio Health Center, situated in Kamaxaqueni Municipal District, Maputo City, served as the control site. Both facilities are part of the National Health Service and routinely provide maternal and child health services, including growth monitoring, immunization and nutrition counseling. The intervention was embedded within routine healthy child consultations and delivered through existing health service platforms.

The intervention period took place between February 2024 and March 2025.

### Participants

The study population comprised caregiver–child pairs, including primary caregivers of infants aged 6–8 months attending routine healthy child consultations at the selected health centers. Caregivers were eligible to participate if they provided written informed consent.

The present analysis focuses exclusively on caregivers' KAP related to infant nutrition, complementary feeding and MNP use. Caregivers completed the structured KAP questionnaire at baseline and at the end of the 6-month follow-up period.

Caregivers of infants who did not meet the eligibility criteria of the parent study were not included in the KAP analysis.

### Sample size

The planned sample size for the parent study was 506 caregiver–child pairs, calculated using G^*^Power version 3.1.9.7 to detect a 0.20 kg between-group difference in child weight, assuming a standard deviation of 0.80 kg, a two-sided significance level of 5% (α = 0.05) and 80% statistical power, as described in the baseline paper (16). The present manuscript reports a secondary analysis focusing on caregivers' KAP, conducted within the same intervention framework and sample. For the intervention phase, 496 caregiver–child pairs were screened for eligibility and 466 were enrolled at baseline (224 at Matola C Health Center and 242 at 1° de Maio Health Center). After attrition during follow-up (9 in the intervention group and 15 in the control group), the final analytical sample for the KAP analysis comprised 442 caregivers (215 intervention; 227 control).

### Recruitment and follow-up

Caregivers were recruited consecutively during routine healthy child consultations at the two study sites. Allocation to the intervention or control group was based on the health center attended and was therefore non-randomized.

**Figure 1:**
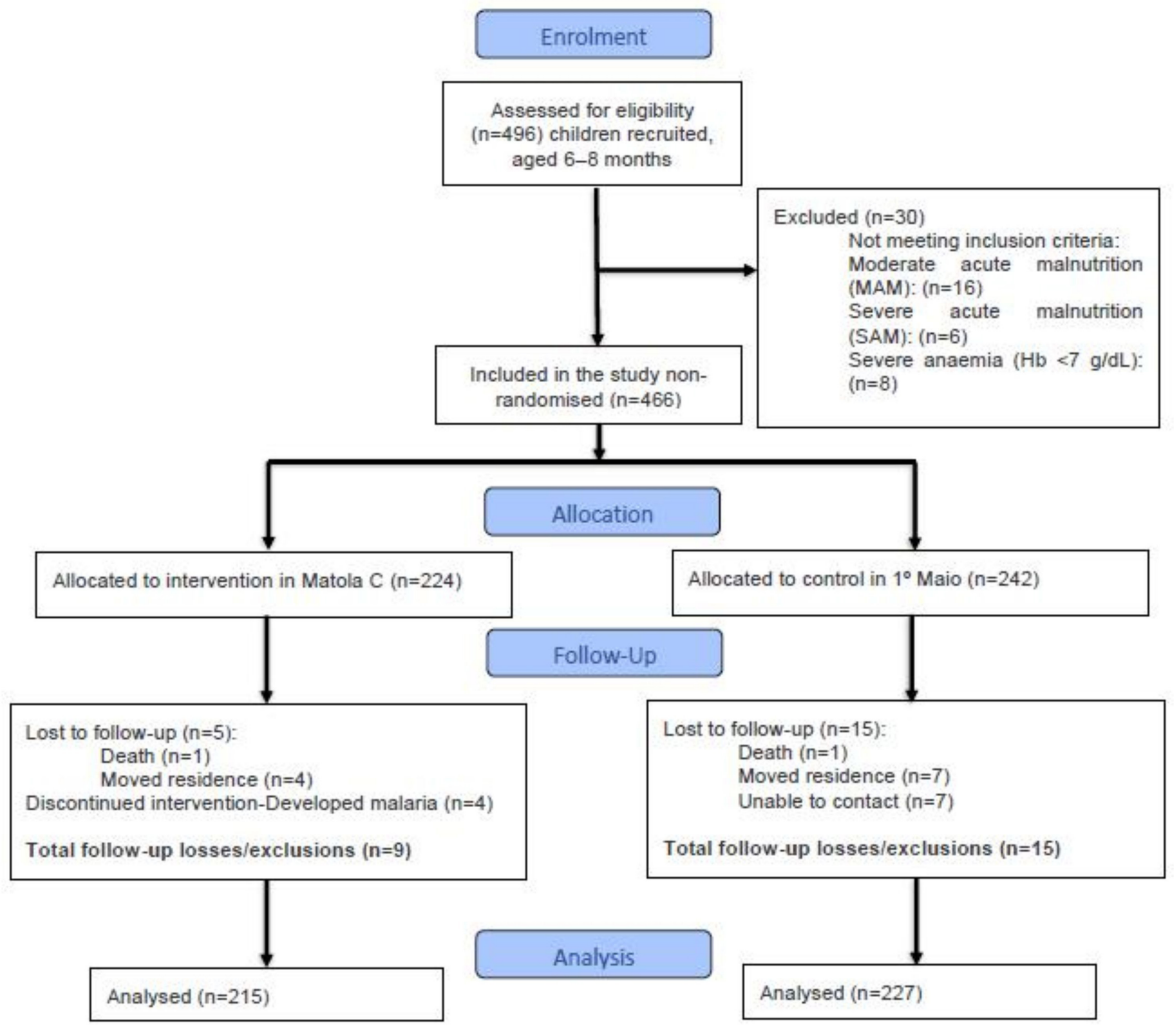
Flow diagram of participant recruitment, follow-up, and analysis.

Caregivers completed the KAP questionnaire at two time points: at baseline, prior to exposure to the intervention, and at the end of the 6-month follow-up period. Follow-up was conducted through routine service contacts. Losses to follow-up were recorded and are presented in the participant flow diagram (Figure 1).

Reporting of this study follows the TREND (Transparent Reporting of Evaluations with Nonrandomized Designs) statement for nonrandomized evaluations.

### Intervention

The intervention consisted of a nutrition education programme delivered through routine child health services. Educational activities focused on infant nutrition, appropriate complementary feeding practices, and MNP use, in accordance with national IYCF guidelines and social and behavior change communication (SBCC) strategies.

Education sessions were delivered by trained health professionals during routine consultations and reinforced during subsequent visits. The intervention aimed to improve caregivers' understanding of recommended practices, strengthen positive attitudes, and support the adoption of appropriate feeding and supplementation behaviors.

### Variables (outcomes)

Primary outcomes were caregivers' KAP related to infant nutrition, complementary feeding and MNP use.

Specifically, the outcomes included:

- Knowledge was measured using a composite score derived from multiple questionnaire items reflecting caregivers' understanding of recommended infant nutrition, complementary feeding and supplementation practices.
- Attitudes were assessed at the item level as binary indicators reflecting agreement with recommended infant feeding and supplementation statements.
- MNP-related practices reflected self-reported behaviors related to the use of micronutrient powder and were assessed descriptively at endline in the intervention group only, since MNP distribution was implemented exclusively within the intervention group.

Sociodemographic and socioeconomic variables (baseline):

Baseline sociodemographic and socioeconomic variables were collected to characterize the caregiver population and to assess baseline comparability between study groups. These included caregiver age, marital status, educational attainment, and monthly household income.

### Instruments and procedures

Data collection was conducted by a trained team of healthcare professionals and project researchers through face-to-face interviews with caregivers. All interviews were conducted in private consultation rooms at the health centers to ensure participants' privacy, comfort, and confidentiality.

A structured questionnaire was specifically developed for this study based on national IYCF guidelines and the study objectives, with the aim of assessing caregivers' KAP related to infant nutrition, complementary feeding and MNP use. The questionnaire was administered at baseline and at the end of the 6-month follow-up period. The full questionnaire is provided as Supplementary File 1.

### Procedures

Before data collection, all members of the field team received standardized training on questionnaire administration, interview techniques, ethical conduct, and procedures to ensure data quality and consistency. Training emphasized neutral interviewing, accurate recording of responses, and respect for participant confidentiality.

Data collection was conducted in two structured phases:

**Phase 1—baseline assessment:**

- At baseline, primary caregivers were interviewed using a structured questionnaire assessing KAP related to infant nutrition, complementary feeding and MNP use. Sociodemographic and socioeconomic data were also collected at this stage.

**Phase 2—endline assessment:**

- At the end of the 6-month intervention period, caregivers were re-interviewed using the same structured questionnaire to assess changes in KAP following exposure to the nutrition education intervention.

## Data analysis

Descriptive statistics were used to characterize caregivers' baseline sociodemographic and economic variables, as well as knowledge, attitudinal and practice outcomes. Continuous variables were summarized as means and standard deviations (SD) when normally distributed, and as medians and interquartile ranges (IQR) when normality assumptions were not met.

Distributional assumptions for knowledge scores were assessed using the Shapiro–Wilk test and examination of skewness and kurtosis values.

Baseline comparability between the intervention and control groups was assessed using independent *t*-tests for continuous variables and chi-squared (χ^2^) tests for categorical variables. When normality assumptions were not met, Mann–Whitney U tests were applied.

Within-group changes in knowledge scores from baseline to endline were analyzed using the Wilcoxon signed-rank test.

The primary intervention effect on knowledge was estimated using difference-in-differences (DiD) analysis to compare changes over time between the intervention and control groups.

Attitudinal indicators were analyzed as proportions at baseline and endline. Changes within groups were assessed as differences in proportions, and DiD estimates were calculated for each attitudinal item to compare changes between groups over time.

Missing data were handled using a complete-case approach. A significance level of 5% was adopted for all analyses. Statistical analyses were performed using SPSS software, version 31.

## Results

### Baseline sociodemographic and socioeconomic characteristics of caregivers

A total of 466 caregivers were included in the baseline analysis, with 224 in the intervention group and 242 in the control group. The mean age of caregivers was similar between the intervention and control groups (27.1 ± 6.6 vs. 27.5 ± 7.5 years; *p* = 0.56). There were no statistically significant differences between groups at baseline in caregiver educational attainment or marital status (*p* > 0.05); however, household income distribution differed between groups (*p* = 0.040) (Table 1).

**Table 1:** Baseline sociodemographic and socioeconomic characteristics of caregivers by study group.

Variable	Intervention (*n* = 224)	Control (*n* = 242)	*p*-value
Age of caregiver (years), mean ± SD	27.1 ± 6.6	27.5 ± 7.5	0.56
**Caregiver's education**, ***n*** **(%)**			
No formal education	5 (2.2)	7 (2.9)	0.897
Primary education	41 (18.3)	47 (19.4)	
Secondary education	160 (71.4)	172 (71.1)	
Higher education	18 (8.0)	16 (6.6)	
**Marital status**, ***n*** **(%)**			
Single	35 (15.6)	51 (21.1)	0.232
Married/union	187 (83.5)	188 (77.7)	
Divorced/separated	1 (0.4)	3 (1.2)	
Widowed	1 (0.4)	0 (0.0)	
**Household monthly income**, ***n*** **(%)**			
< Minimum wage (8 758 MZN)	97 (43.3)	98 (40.5)	0.040
= Minimum wage (8 758 MZN)	45 (20.1)	76 (31.4)	
>Min. wage up to 10 000 MZN	34 (15.2)	38 (15.7)	
>10 000 MZN	48 (21.4)	30 (12.4)	

Age was compared using an independent samples *t*-test. Categorical variables were compared using Pearson's chi-square test or Fisher's exact test, as appropriate.

### Baseline knowledge of caregivers by study group

At baseline, caregivers' knowledge scores differed significantly between the intervention and control groups (Table 2). Knowledge scores were higher in the intervention group (median: 75%; IQR: 50–75) compared to the control group (median: 50%; IQR: 50–75), with statistically significant differences (Mann–Whitney U test, *p* < 0.001).

**Table 2:** Baseline knowledge of caregivers by study group.

Outcome (baseline)	Intervention [median (IQR)]	Control [median (IQR)]	*p*-value
Knowledge score (%)	75 (50–75)	50 (50–75)	< 0.001

Data are presented as median and interquartile range (IQR). Between-group comparisons were performed using the Mann–Whitney U test.

### Changes in caregivers' knowledge after the intervention

Caregivers' knowledge scores increased significantly from baseline to endline in both study groups (Table 3). Table 3 includes only participants with complete baseline and endline data (complete-case analysis). In the intervention group, knowledge scores increased from a median of 75% (IQR: 50–75) at baseline to 100% (IQR: 100–100) at endline (Wilcoxon signed-rank test, *p* < 0.001). Similarly, in the control group, scores increased from 50% (IQR: 50–75) to 80% (IQR: 80–100) (p < 0.001).

**Table 3:** Changes in caregivers' knowledge after the intervention.

Outcome	Intervention baseline [median (IQR)]	Intervention endline [median (IQR)]	*p* (within)	Control baseline [median (IQR)]	Control endline [median (IQR)]	*p* (within)	DiD (95% CI)	*p* (did)
Knowledge score (%)	75 (50–75)	100 (100–100)	< 0.001	50 (50–75)	80 (80–100)	< 0.001	+1.8 (−2.3 to 5.9)	0.381

Data are presented as median and interquartile range (IQR). Within-group comparisons were performed using the Wilcoxon signed-rank test. Between-group comparisons were performed using the Mann–Whitney U test. Difference-in-differences (DiD) estimates compare changes over time between groups. In this table includes only participants with complete baseline and endline data (complete-case analysis).

At endline, caregivers in the intervention group had significantly higher knowledge scores than those in the control group (Mann–Whitney U test, *p* < 0.001).

Difference-in-differences analysis indicated that the magnitude of improvement did not differ significantly between groups (DiD = +1.8 pp, 95% CI −2.3 to 5.9; *p* = 0.381). Distributional diagnostics are presented in Supplementary Table S1.

### Changes in caregivers' attitudinal indicators after intervention

Changes in attitudinal indicators are presented in Table 4. In the intervention group, improvements were observed across all key indicators between baseline and endline. The proportion of caregivers supporting exclusive breastfeeding until 6 months increased from 81.9% to 95.3% (+13.4 pp), while agreement with avoiding water or other liquids before 6 months increased from 69.8% to 81.4% (+11.6 pp). Support for MNP use increased from 90.0% to 97.6% (+7.6 pp), and reported participation in nutrition education activities was already high at baseline (97.7%) and reached 100% at endline.

**Table 4:** Changes in caregivers' attitudinal indicators by study group.

Attitudinal indicator	Intervention baseline % (*n*/*N*)	Intervention endline % (*n*/*N*)	ΔI (pp)	Control baseline % (*n*/*N*)	Control endline % (*n*/*N*)	ΔC (pp)	DiD (pp)	*p* (DiD)
Exclusive breastfeeding until 6 months	81.9 (176/215)	95.3 (205/215)	+13.4	90.7 (204/225)	93.3 (210/225)	+2.6	+10.8	< 0.001
No water/other liquids before 6 months	69.8 (150/215)	81.4 (175/215)	+11.6	81.0 (183/226)	38.5 (87/226)	−42.5	+54.1	< 0.001
Support for MNP use	90.0 (188/209)	97.6 (204/209)	+7.6	79.3 (180/227)	24.2 (55/227)	−55.1	+62.7	< 0.001
Participation in nutrition education activities	97.7 (209/214)	100.0 (214/214)	+2.3	95.2 (216/227)	66.5 (151/227)	−28.7	+31.0	< 0.001

Values represent proportions of caregivers providing responses consistent with recommended practices. Δ indicates change in pp from baseline to endline. DID estimates reflect between-group differences in changes from baseline to endline.

In contrast, the control group showed modest improvement in exclusive breastfeeding (+2.6 pp), but declines were observed in several other attitudinal indicators. Agreement with avoiding pre-lacteal liquids decreased from 81.0% at baseline to 38.5% at endline (−42.5 pp), support for MNP use decreased from 79.3 to 24.2% (−55.1 pp), and reported participation in nutrition education activities decreased from 95.2 to 66.5% (−28.7 pp).Difference-in-differences analyses indicated statistically significant effects favoring the intervention group across all attitudinal indicators, with DiD estimates ranging from +10.8 to +62.7 pp (all *p* < 0.001).

### Caregivers' practices related to MNP use at endline (intervention group)

At baseline, no caregiver reported offering MNP. At endline, all caregivers with valid responses in the intervention group reported offering MNP to their children (Table 5). Most caregivers administered MNP once daily (79.5%). Regarding preparation practices, 76.7% of caregivers reported mixing MNP with food after cooling, which is considered correct practice, while 23.3% reported mixing MNP with hot food, representing incorrect practice.

**Table 5:** Caregivers' practices related to MNP use at endline (intervention group).

MNP-related practice	*n*	%
Offered MNP to the child^*^	215	100.0
**Frequency of MNP administration (*****n*** = **215)**		
Once per day	171	79.5
More than once per day	35	16.3
Not every day	9	4.2
**Method of MNP administration (*****n*** = **215)**		
Mixed with food after cooling (correct practice)	165	76.7
Mixed with hot food (incorrect practice)	50	23.3

Percentages are based on valid responses among caregivers in the intervention group at endline. MNP-related practices were not applicable at baseline or in the control group. ^*^Assessed only at endline in the intervention group.

## Discussion

At baseline, the intervention and control groups were comparable in caregiver age, educational attainment and marital status, a pattern consistent with findings from urban and peri-urban nutrition studies in sub-Saharan Africa, including Uganda, Ethiopia and South Africa, where caregivers accessing public health facilities tend to show similar demographic profiles and levels of formal education (1, 5, 6). Educational attainment has been consistently associated with caregivers' IYCF knowledge and attitudes in studies conducted in Uganda, India and Malaysia (6, 7, 17, 18). The absence of baseline differences in education between groups suggests that subsequent changes in caregivers' knowledge and attitudes were unlikely to be driven by pre-existing disparities in schooling.

In contrast, household income differed significantly between groups at baseline. Evidence from Demographic and Health Surveys in Mozambique and other low- and middle-income countries shows that household income is strongly associated with dietary diversity, food security and caregivers' ability to translate nutrition knowledge into practice (3, 12, 16, 19). These findings highlight the presence of socioeconomic heterogeneity even within urban populations with relatively good access to health services, a pattern also documented in the studies cited above.

Variability in baseline IYCF knowledge across health facilities has been widely reported across sub-Saharan Africa, including studies from Uganda, Ethiopia and South Africa, where differences at enrolment are often linked to the frequency and quality of previous exposure to nutrition education and counseling rather than formal education alone (1, 5, 6). Evidence from nutrition education and KAP studies conducted in India and Malaysia further suggests that caregivers' attitudes toward infant feeding may be influenced by multiple contextual factors, including cultural norms and household constraints (8, 17, 18).

Practices related to MNP use were not applicable at baseline, as MNP had not yet been introduced to caregivers. At endline, although uptake was universal in the intervention group, mixing MNP with hot food remained a suboptimal preparation practice, suggesting that correct use may still be influenced by practical constraints and the need for continued counseling. Similar implementation challenges have been reported in MNP programmes in Burkina Faso, Ghana, Rwanda and Madagascar, reinforcing the importance of combining supplementation with sustained behavior-change support and context-adapted guidance (2, 20–22).

The substantial improvement in caregivers' knowledge observed in both study groups indicates that knowledge related to IYCF is highly responsive to repeated exposure to nutrition messages delivered through routine health services. This finding is consistent with evidence from Uganda, Ethiopia and South Africa, where routine facility-based counseling has been shown to improve caregiver knowledge over time, particularly among caregivers who attend child health services regularly during infancy (1, 5, 6). The absence of a statistically significant difference in knowledge gains between the intervention and control groups suggests that, in this urban setting, routine services alone may be sufficient to substantially improve caregiver knowledge. Similar patterns have been reported in studies from India and Malaysia, where improvements in caregiver knowledge were observed in both intervention and comparison groups receiving consistent facility-based counseling (7, 17, 18). Nevertheless, the higher endline knowledge levels observed in the intervention group suggest that structured and reinforced education may have contributed to greater consistency and completeness of caregivers' knowledge, particularly given that counseling in this group was regularly reinforced by the study nutrition team through repeated contacts during routine health consultations.

Beyond knowledge acquisition, the intervention also influenced caregivers' attitudes toward recommended infant feeding practices. Attitudinal indicators showed a clear pattern favoring the intervention group, with improvements observed across key indicators including support for exclusive breastfeeding until 6 months, avoidance of water or other liquids before 6 months, acceptance of micronutrient powder use, and participation in nutrition education activities. In contrast, several attitudinal indicators deteriorated in the control group over the same period. Similar findings have been reported in intervention studies conducted in different settings. For example, a quasi-experimental study in Bangladesh demonstrated significant improvements in mothers' attitudes toward complementary feeding following a structured nutrition education intervention. Comparable improvements in caregivers' attitudes and feeding behaviors have also been documented in studies conducted in Ethiopia, Uganda and India following exposure to nutrition education and behavior change communication strategies (5, 6, 18, 23). These findings suggest that reinforced counseling delivered through routine health services can play an important role in strengthening caregivers' attitudes toward recommended infant feeding and supplementation practices.

The universal uptake of MNP among caregivers in the intervention group and the high adherence to daily administration indicate strong acceptability and feasibility of home fortification when MNP distribution is combined with structured counseling delivered through routine child health consultations. Similar levels of uptake and regular use have been reported in facility-based MNP programmes in Brazil and Ghana, where integration within routine child health services and repeated caregiver counseling were key determinants of adherence (24–26). These findings support WHO guidance and Mozambican national strategies on IYCF and SBCC, which recommend strengthening nutrition education with practical interventions, such as MNP supplementation, delivered through routine health services and supported by sustained counseling (9, 13, 14, 27).

## Conclusion

Strengthening nutrition education delivered through routine child health services was associated with substantial improvements in caregivers' knowledge related to infant feeding and MNP use in this urban setting. Although knowledge increased in both study groups, reinforced and structured counseling contributed to higher endline knowledge levels in the intervention group. The intervention was also associated with improvements in caregivers' attitudes toward recommended infant feeding practices, highlighting the importance of sustained counseling and behavior change communication within routine services. Universal uptake and high adherence to MNP use in the intervention group further demonstrate that home fortification, when supported by reinforced counseling within routine services, is highly acceptable and feasible within routine health systems. These findings support strengthening and optimizing existing child health programmes to promote recommended feeding practices and reduce micronutrient deficiencies, in line with WHO guidance and Mozambican national nutrition strategies.

## Study limitations

This study has some limitations that should be considered when interpreting the findings. Caregivers' knowledge and attitudes were assessed using self-reported questionnaire responses, which may be subject to social desirability bias, particularly at endline following repeated exposure to counseling.

In addition, practices were assessed only in relation to MNP use and only at endline in the intervention group. Infant feeding practices were not reassessed at endline because questions related to complementary feeding timing, food introduction and food types referred to behaviors that had already occurred and were therefore captured at baseline and analyzed separately in a previous publication. Consequently, this study focused on changes in caregivers' knowledge, attitudes and MNP-related practices rather than broader infant feeding behaviors over time.

## Data Availability

The raw data supporting the conclusions of this article will be made available by the authors, without undue reservation.
