# Supplementary material for: Effect of a nutrition education intervention on caregivers' knowledge, attitudes and practices regarding infant feeding and micronutrient powder use in urban health centers in Maputo, Mozambique
Source: Front Nutr. 2026 Jun 2;13:1833701. doi: 10.3389/fnut.2026.1833701 (PMC13270930; doi:10.3389/fnut.2026.1833701)
Supplement: Supplementary file 1 [file Table_1.docx]

**Supplementary Table S1. Assessment of distributional assumptions and sensitivity analyses for knowledge scores**

| **Variable** | **Group** | **N** | **Skewness** | **Kurtosis** | **Shapiro–Wilk (p)** | **t-test (p)** | **Mann–Whitney U (p)** |
| --- | --- | --- | --- | --- | --- | --- | --- |
| Knowledge (Baseline) | Intervention | 224 | −1.099 | 1.726 | <0.001 | <0.001 | <0.001 |
| Knowledge (Baseline) | Control | 242 | −0.265 | −0.163 | <0.001 | — | — |
| Knowledge (Endline) | Intervention | 215 | −2.490 | 5.565 | <0.001 | <0.001 | <0.001 |
| Knowledge (Endline) | Control | 227 | 0.342 | −0.119 | <0.001 | — | — |
| Knowledge Change Score | Intervention | 215 | 0.922 | 1.610 | <0.001 | 0.381 | 0.650 |
| Knowledge Change Score | Control | 227 | 0.256 | −0.228 | <0.001 | — | — |
